# Role of Transcription Factors in Steatohepatitis and Hypertension after Ethanol: The Epicenter of Metabolism

**DOI:** 10.3390/biom6030029

**Published:** 2016-06-24

**Authors:** Rais A. Ansari, Kazim Husain, Syed A. A. Rizvi

**Affiliations:** 1 Department of Pharmaceutical Sciences, College of Pharmacy, Health Professions Division, Nova Southeastern University, 3200 S University Drive, Fort Lauderdale, FL 33328, USA; srizvi@nova.edu; 2 Department of Physiology, Pharmacology and Toxicology, Ponce School of Medicine, P.O. Box 7004, Ponce, PR 00732-2575, USA; kazimhusain@hotmail.com

**Keywords:** ethanol, alcoholic liver disease, steatohepatitis, hypertension, transcription factor, gene regulation

## Abstract

Chronic alcohol consumption induces multi-organ damage, including alcoholic liver disease (ALD), pancreatitis and hypertension. Ethanol and ethanol metabolic products play a significant role in the manifestation of its toxicity. Ethanol metabolizes to acetaldehyde and produces reduced nicotinamide adenine dinucleotide (NADH) by cytosolic alcohol dehydrogenase. Ethanol metabolism mediated by cytochrome-P450 2E1 causes oxidative stress due to increased production of reactive oxygen species (ROS). Acetaldehyde, increased redox cellular state and ROS activate transcription factors, which in turn activate genes for lipid biosynthesis and offer protection of hepatocytes from alcohol toxicity. Sterol regulatory element binding proteins (SREBPs) and peroxisome proliferator activated-receptors (PPARs) are two key lipogenic transcription factors implicated in the development of fatty liver in alcoholic and non-alcoholic steatohepatitis. SREBP-1 is activated in the livers of chronic ethanol abusers. An increase in ROS activates nuclear factor erythroid-2-related factor-2 (Nrf2) and hypoxia inducible factor (HIF) to provide protection to hepatocytes from ethanol toxicity. Under ethanol exposure, due to increased gut permeability, there is release of gram-negative bacteria-derived lipopolysaccharide (LPS) from intestine causing activation of immune response. In addition, the metabolic product, acetaldehyde, modifies the proteins in hepatocyte, which become antigens inviting auto-immune response. LPS activates macrophages, especially the liver resident macrophages, Kupffer cells. These Kupffer cells and circulating macrophages secrete various cytokines. The level of tumor necrosis factor-α (TNFα), interleukin-1beta (IL-1β), IL-6, IL-8 and IL-12 have been found elevated among chronic alcoholics. In addition to elevation of these cytokines, the peripheral iron (Fe^2+^) is also mobilized. An increased level of hepatic iron has been observed among alcoholics. Increased ROS, IL-1β, acetaldehyde, and increased hepatic iron, all activate nuclear factor-kappa B (NF-κB) transcription factor. Resolution of increased reactive oxygen species requires increased expression of genes responsible for dismutation of increased ROS which is partially achieved by IL-6 mediated activation of signal transducers and activators of transcription 3 (STAT3). In addition to these transcription factors, activator protein-1 may also be activated in hepatocytes due to its association with resolution of increased ROS. These transcription factors are central to alcohol-mediated hepatotoxicity.

## 1. Introduction

Ethanol or ethyl alcohol (C_2_H_5_OH) is a fermentation product of grain, fruit juice and honey, etc. and has been used by humans for a long time. In fact, early Egyptians, Chinese in around 7000 BC, Indian between 3000 and 2000 BC, Babylonians as early as 2700 BC and Greeks and South Americans have used fermented beverages and alcoholic drinks [1]. Spirits, a form of alcohol containing methanol and iron, were used largely for medicinal purposes in sixteenth century [2]. In the UK, at the beginning of the eighteenth century, spirits were heavily used. During the nineteenth century, a change in practice led to a movement away from heavy ethanol dependence toward moderate application of ethanol. In 1920, the US passed a law banning manufacturing, sale, import and export of intoxicating liquors. A divergent view on the beneficial and harmful effects of ethanol use exists among scientist and researchers. Current and past research suggests that moderate alcohol intake is beneficial to the cardiovascular system and lowers blood pressure while excessive use is harmful to the cardiovascular system and causes elevation in blood pressure. Low to moderate alcohol use has been linked to decreased incidence of coronary heart disease and increase in longevity [3,4]. It has been used as an analgesic and widely available to individuals in pain [2,5]. Experiments with rats in preclinical studies showed a decrease in systolic blood pressure of 1.0 g/Kg for 12 weeks [6]. Moderate drinking is defined as two drinks per day for younger men, and one drink for men over 65 and women of all ages. As an example, a drink is defined as 12 ounces, i.e., 355 millimeters of beer, 5 ounces or 148 milliliters of wine or 1.5 ounces (44 mL) of 80-proof distilled spirits.

Today, alcohol and alcoholic beverages are used more often and have become part of human society. Due to its easy availability, excessive and chronic use has created major public health crises worldwide. In US alone, alcohol abuse affects more than 20 million individuals and causes over 100,000 deaths annually [7,8]. Chronic ethanol usage causes liver, gastrointestinal, nervous and cardiovascular toxicities [9]. In this review, we will focus our attention on alcoholic liver disease and elevation in blood pressure with emphasis on the ethanol [3,4] metabolism and activation of transcription factors induced steatosis and steatohepatitis. Additionally, we will focus on alcohol-mediated hypertension especially of the renin-angiotensin-aldosterone system (RAAS), which is reviewed in detail by K. Husain et al. [10].

## 2. Ethanol-Mediated Hepatosteatosis, Steatohepatitis and Role of Transcription Factors

Fatty liver (steatosis) is the earliest, most common response of the liver to moderate or large doses of alcohol, including both binge drinking and chronic alcohol abuse [11]. Hepatic fatty acid and triglycerides are increased after acute and chronic ethanol consumption [12]. Utilizing a cell-based model, increased lipogenesis has been shown to occur due to higher expression and activation of lipogenic enzymes, including fatty acid synthase (FAS), acyl CoA carboxylase (ACC), ATP citrate lyase (ACL), stearyl CoA desaturase and malic enzymes [13,14]. The lipogenic enzymes are under regulation of a transcription factor, sterol regulatory element binding protein (SREBP). SREBPs are a class of transcription factors which are involved in fat and cholesterol regulation [15]. There are three isoforms of SREBPs, SREBP-1a, SREBP-1c and SREBP-2. SREBP-1a and 1c are an alternatively spliced form of SREBP-1 gene. Both SREBP-1a and 1c are involved in regulation of fatty acid biosynthesis [16]. SREBPs exist as the precursor molecules in endoplasmic reticulum and are activated by SREBP cleavage activating protein (SCAP) and site-1 protease (S1P) and site-2 protease (S2P) and their activation is dependent on the cellular cholesterol level [16]. Recent work has shown that ethanol exposure to cultured hepatocytes leads to increased levels of the active/nuclear (n) form of SREBP-1 (nSREBP-1) [17]. The SREBP regulatory loop is disturbed in the event of ethanol exposure and is dependent on alcohol metabolism. The metabolic product, acetaldehyde, is capable of activating SREBP-1 as inhibitors of alcohol dehydrogenase (4-methylpyrazole) inhibited the SREBP activation in cultured rat hepatocytes and HepG2 cells [18,19]. The SREBP-1c level is increased transcriptionally and post-translationally by decreased proteasomal degradation of the protein in acute and chronic alcohol exposure [20]. Decreased alcoholic steatosis has been observed in SREBP-1c null mice [21].

Peroxisome proliferator activated-receptors (PPARs) are a member of the nuclear receptor superfamily. The PPAR subfamily consists of three isotypes, PPARα, PPARγ and PPARβ/δ. PPARs are important players in fatty acid metabolism, lipogenesis and adipogenesis [22]. Ethanol exposure not only inhibits the transcriptional activity of PPARα but also decreases the DNA binding activity of PPARα. Utilizing hepatoma cells or primary hepatocytes and selective inhibitors of ethanol metabolizing enzymes, it was shown that the effects of ethanol were dependent on ethanol metabolizing enzymes [23]. These findings were further validated in chronic ethanol feeding studies in animals [24]. PPARs bind to DNA as an obligate heteromer with retinoid X receptor (RXR: PPARα) and the level of RXR is decreased with ethanol exposure and hence the transcriptional activity of PPARα [25] is also suppressed.

PPARγ is expressed as two major isoforms, γ1 and γ2, which are generated from the same gene by alternative splicing [26]. PPARγ participates in the transcriptional activation of numerous adipogenic and lipogenic genes. The latter is important for adipocyte maturation, lipid accumulation and insulin sensitive glucose transporter. SREBP-1c enhances the transcriptional activity of PPAR-γ. SREBP-1c increases PPARγ activity either by increasing cellular levels of ligand or directly activating the transcription factor [27,28]. With chronic alcohol exposure or exposure with large dose(s) of alcohol, the level of SREBP is increased [16]. Therefore, SREBP activity with ethanol toxicity is linked with the activity of PPARγ. Reports on the effects of ethanol on the activity and expression of PPARγ are lacking. It has been established that the increased expression of PPARγ results in development of fatty liver, which is typical for chronic alcohol abuse. Expression of PPARγ is increased in fatty livers of several animal models of obesity and diabetes. PPARγ1 is expressed in liver, adipose and other tissues. The function of PPARγ1 and PPARγ2 in mediating the liver steatosis has been deciphered by employing the protein overexpression methodology. The high levels of PPARγ1 expression in mouse liver are sufficient for adipogenic transformation and expression of adipogenic markers in hepatocytes. Increased PPARγ activity can lead to the development of fatty liver [29]. In addition, the role of PPARγ1 in hepatic steatosis development was confirmed by overexpression of PPARγ1 in PPARα−/− transgenic mice [30]. Furthermore, the role of PPARγ2 in liver steatosis has also been determined by employing the cell-based overexpression technology. Utilizing immortalized hepatocytes (AML-12), which overexpress human transforming growth factor-α, PPARγ2 selectively upregulated adipogenic genes in hepatocytes but failed to induce a complete adipogenic program. Thus it was concluded that PPARγ2 is responsible for tissue-specific regulation of lipid homeostasis and pathology [31]. Moreover, PPARγ directly contributes to hepatic steatosis acting either down-stream or in parallel with SREBP-1 [32]. SREBP-1c in cooperation with PPARγ2 may stimulate fatty acid synthesis, thus further contributing to adipogenecity [33]. More importantly, CAAT enhancer binding protein-α (C/EBPα), related to leucine zipper transcription factors also promotes adipogenesis and its effect is dependent on PPARγ [34]. SREBP-1c, PPARγ2 and C/EBPα contain their own response elements in their respective promoters and are autoinduced [35].

In addition to its role in adipogenesis, the increased levels of PPARγ have been observed in inflammation [36], which is a typical condition of alcohol toxicity and type 2 diabetes. Treatment of type 2 diabetic patients with a PPARγ agonist reduces the inflammation due to increased insensitivity to insulin. However, the direct effects of ethanol exposure on PPARγ and PPARδ have not been demonstrated despite reduction in insulin action by ethanol exposure having been observed to link insulin insensitivity and resistance in type2 diabetes (T2DB) [37]. In a report, ethanol mediated hepatic toxicity was antagonized by treatment of animals with PPARγ agonist, pioglitazone (500 µg/kg) each day for 8 weeks [38]. In this study, pioglitazone was able to reverse all the parameters of alcohol liver toxicity except gut permeability. These data implicate PPARγ in not only protection of hepatotoxicity (antagonizing NF-κB activation both by cytokines and ROS, a fact stated later) but also as preventive of ethanol-mediated hepatosteatosis.

Alcohol exposure not only leads to steatosis but proceeds to steatohepatitis and fibrogenesis. The fibrotic transformation of liver occurs after death of hepatocytes and deposition of collagen and the collagen is secreted by hepatic stellate cells, which are activated or transdifferentiated. This transdifferentiation occurs due to loss of adipogenic program in stellate cells. The transcription factors (C/EBPs, PPARs and SREBP-1c and LXRα), which cause lipid accumulation in hepatocytes are important for maintenance of quiescent state of hepatic stellate cells. The adipogenic regulation of hepatic stellate cells is downregulated causing fatty liver and liver fibrosis [35].

**Figure 1:**
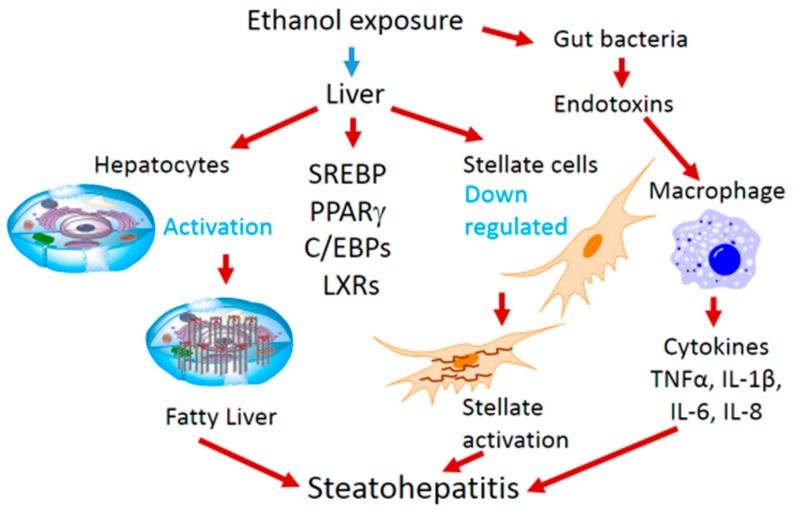
The role of transcription factors in hepatosteatosis and steatohepatitis.

Interaction of parenchymal cells and liver immune cells, which are Kupffer cells, play a significant role in hepatitis after steatosis of liver. Increased blood circulating levels of lipopolysaccharide (LPS) have been observed among chronic alcoholics. The source of LPS is the gut, where bacteria are killed due to ethanol. Alcohol liver disease (ALD) bears characteristics of chronic, low-level inflammation of the liver. Alcohol exposure primes the activation of peripheral monocytes of alcoholic hepatitis patients; they produce more pro-inflammatory cytokines in response to LPS [39]. Hepatic inflammation involves both resident Kupffer cells and recruited inflammatory cells which could be neutrophils and lymphocytes. LPS activates Kupffer cells resulting into production of tumor necrosis factor-α (TNFα) and other inflammatory cytokines [40]. The level of TNFα is increased in chronic alcoholics and in a mouse chronic alcohol exposure model [39,41]. In addition to Kupffer cells, natural killer cells also contribute to liver inflammation and fibrosis as hepatic stellate cell killing is reduced by NK cells after alcohol exposure [42]. A summary of alcohol-mediated hepatosteatosis and steatohepatitis involving various factors is depicted in Figure 1.

## 3. Ethanol Mediated Effects on Transcription Factors

Studies have demonstrated that adenosine monophosphate-activated protein kinase (AMPK) is centrally involved in the regulation of hepatic triglycerides, cholesterol and fatty acid biosynthesis, which is under the transcriptional regulation of PPARα and SREBP-1. It has been established that SREBP-1 is the target of AMPK [16]. Activation of AMPK in hepatocytes decreases nSREBP-1 levels due to enhanced proteasomal degradation [43]. Metformin and aminoimidazole-4-carboxamide ribonucleotide (AICAR) are known activators of AMPK. Treatment of hepatoma cells with metformin and AICAR causes decreased nSREBP, while ethanol treatment was able to reverse the effects [43]. Therefore, AMPK can play a significant role in ethanol mediated effects on hepatic SREBP-1. This finding has been further substantiated by a report that a fat-derived hormone, adiponectin, which is upregulated during ethanol exposure, suppresses AMPK, thereby alleviating alcoholic fatty liver disease [44]. In addition, ER stress also activates SREBP [45]. Alcohol is known to produce ER-stress, which relates to unfolded protein response [46]. Cell based models with HepG2 and VL-17A (constitutively expressing mouse alcohol dehydrogenase) when exposed to alcohol results in ER fragmentation and incomplete ER stress [47]. In addition, increased homocysteine has also been implicated in ER-stress [48]. The transcriptional activity of SREBP-1c is inhibited by AMPK, which phosphorylates SREBP-1c at serine372. Due to reduced AMPK activity in ethanol toxicity, the transcriptional activity of SREBP-1c would be elevated, causing hepatosteatosis [49]. Another gene product, lipin-1 is involved in triglyceride synthesis and fatty acid oxidation, and the upregulation of lipin-1 has been observed in ethanol mediated fatty liver and this regulation remains under AMPK. When AMPK was either activated or constitutively active AMPK was expressed, the lipin-1 gene activity was inhibited. Studies demonstrated that lipin-1 gene activation was mediated through AMPK inhibition and SREBP activation [50]. Activation of SREBP has also been observed with administration of TNF-α and the level of TNF-α is increased due to increased LPS level in circulation after ethanol exposure. TNF-α stimulates the production of active nSREBP in human hepatocytes. TNF-α also upregulates the mRNA level of SREBP in rat livers [51,52]. The metabolic product, acetaldehyde can activate SREBP [18]. Thus SREBP activation is linked to AMPK mediated activation, ER-stress, TNF-α release from Kupffers after LPS mediated activation and alcohol metabolic product, acetaldehyde.

In addition to the regulation of lipogenic genes via SREBP-1, ethanol has been shown to alter liver activating protein (LAP) and liver inhibiting protein (LIP) in rats chronically fed ethanol. Both LAP and LIP are basic helix loop helix transcription factors, which bind to CAAT sequences of DNA and are referred to as CAAT/enhancer binding protein (C/EBP). LAP is a full length C/EBP-β, which activates liver-specific transcription of genes. LIP is the short form of C/EBP-β and inhibits hepatic gene transcription. C/EBP class of transcription factors (α, β, γ, δ and ε) are capable of homo- and heterodimerization and thus control the liver specific gene transcription either by activation or inhibition. C/EBPs are activated in endoplasmic reticulum-stress (ER-Stress) induced by ethanol [53,54]. Chronic ethanol exposure in rats causes increase in LAP (C/EBP-β) and reduction in LIP and this increase in LAP versus LIP is responsible for ethanol induced effects (increase) on Class I alcohol dehydrogenase, an enzyme which is involved in alcohol biotransformation [55].

In addition to ALD, mild to moderate alcohol intake leads to increased hepatic iron-II (Fe^2+^) level [56]. Increased level of Fe^2+^ has been observed in Kupffer cells in an animal model of ALD [57,58]. Fe^2+^ activates nuclear factor-kappa B (NF-κB), which causes the increased biosynthesis of proinflammatory cytokines and tumor necrosis factor-α [57,58,59]. Increased hepatic Fe^2+^ and alcohol metabolism in liver produce ROS and hypoxia [60]. Hypoxic condition and activation of hypoxia inducible transcription factor-1alpha (HIF-1α) has been observed with ethanol in control and an iron sensing peptide, hepcidin, in knockout mice [61,62]. Another way HIF-1α can be induced is by direct ethanol effect on the liver. Chronic ethanol drinking increases oxygen consumption, which results into pericentral hypoxia in the liver. The liver tissue adapts to hypoxia by activating HIFs for regulating glucose uptake and utilization and several other functions [63,64,65]. Several animal studies demonstrate that acute and chronic ethanol exposure leads to HIF activation [64,66,67,68]. After activation, HIF translocates to the nucleus where it activates and/or represses the genes containing the hypoxia response element (HRE) [69]. Fatty acid synthase (FAS) gene is upregulated in alcohol exposure as well as in tumor cells, which have hypoxic microenvironment. The activation of FAS gene by hypoxia requires activation of protein kinase B (Akt)-mediated HIF-1 activation, which further activates SREBP-1 [70].

Peroxisome proliferator-activated receptors (PPARs) are transcription factors of nuclear hormone receptor superfamily and are activated by agonists. PPAR-α can be activated by free fatty acid binding which regulates the genes of free fatty acids oxidation, transport and export [71]. PPAR-α activity is suppressed in hepatocytes and therefore fatty acid oxidation is decreased after alcohol consumption [72]. The oxidative stress produced by CYP2E1 metabolism of ethanol inhibits PPAR-α activity [73]. Acetaldehyde, the metabolic product of ethanol, inhibits the DNA-binding and transcriptional activity of PPAR-α in hepatocytes [23]. Compounds which activate PPAR-α antagonize ethanol mediated PPAR-α dysfunction and hepatic lipid metabolic abnormalities in mice. PPAR-α agonist treatment protects liver from alcohol induced steatosis and inflammation [24,74]. The aforementioned studies confirm the role of PPAR-α in maintenance of lipid homeostasis and development of alcohol induced hepatosteatosis. In addition to PPAR-α, other PPARs like PPAR-γ have emerged as an important player in treatment of type-2-diabetes (T2DB) and PPAR-γ ligands, thiazolidinediones are used clinically for the treatment of T2DB [75]. PPAR-γ forms obligate heteromer with another nuclear receptor, retinoid-X-receptor-α (RXR-α). PPAR-γ/RXR-α bind to the PPAR response element (PPREs) and regulate transcription of genes of inflammation and fatty acid biosynthesis [75]. In addition to heteromerization with RXR-α, PPAR-γ interacts with other transcription factors. PPAR-γ interacts with activator protein (AP-1), STATs (signal transducers and activators of transcription) and NF-κB. PPAR-γ blocks the NF-κB mediated transcriptional activation of pro-inflammatory cytokines, which causes inflammation, a common outcome of ethanol toxicity. PPAR-γ is expressed well in adipose tissue where it is regulating the fatty acid metabolism. PPAR-γ is expressed in liver at only 10%–30% of the level of adipose tissue. Obesity and nutrition can upregulate the PPAR-γ expression in liver [76]. However, the mechanism of this induction is not clear. It has been observed that the level of PPAR-γ is high in livers of murine model of obesity and T2DB [77,78]. Chronic alcohol exposure in rats is reported to decrease the level of PPARγ in liver [79]. PPAR-γ agonists lower blood pressure in human diabetic patients and animal models and this effect is independent of their insulin sensitizing effects [75]. Prevention of ethanol mediated liver injury has been demonstrated in 4-week alcohol exposure to animals by treatment with PPAR-γ agonist. By inhibiting NF-κB, PPAR-γ agonist blocked the cytokine release by Kupffers cells isolated from 4 weeks alcohol-exposed animals [38]. The role of PPAR-γ in relieving the symptoms of T2DB by antagonizing NF-κB is evident but its role in transcriptional regulation of liver specific genes especially of hepatocytes is not fully deciphered which could play a significant role in expression of hepatotoxicity of alcohol.

The role of another transcription factor, carbohydrate response element-binding protein (ChREBP), which controls lipogenesis, has not attracted much attention in alcohol-induced hepatosteatosis. Alcohol has been shown to activate ChREBP in hepatoma cells and C57BL/6J mice exposed to a Liber-DeCarli diet [80]. The activation of ChREBP ocurrs via inhibition of AMP-activated protein kinase (AMPK). The decreased level of AMPK activity results due to increased activity of protein phosphatase 2A (PPA2), which is activated due to xylulose-5-phosphate produced from glucose. PPA2 dephosphorylates ChREBP resulting in translocation from cytoplasm to nucleus [81,82]. Activated ChREBP causes transcriptional activation of lipogenic genes, like ACC and FAS [83]. ChREBP is also activated in high glucose levels observed in T2DB and is involved in conversion of carbohydrate into triglycerides. Its roles in non-alcoholic fatty liver disease (NAFLD), which parallels alcoholic fatty liver, is implicated in transcriptional activation of lipogenic genes, such as FAS, ACC and liver pyruvate kinase [84,85]. Therefore, knockout models of ChREBP exhibit less hepatosteatosis, decreased levels of SCD1, ACC, FAS and liver pyruvate kinase (LPK), suggesting its role in liver steatosis [86,87].

## 4. Ethanol-Mediated Oxidative Stress and Cellular Protection

Ethanol is extensively metabolized to acetaldehyde by the enzyme alcohol dehydrogenase and acetaldehyde is further oxidized to acetate by acetaldehyde dehydrogenase/oxidase in the liver, which may lead to the generation of reactive oxygen species (ROS). On the other hand, chronic high-dose ethanol ingestion induces hepatic microsomal cytochrome P450 II E1 leading to generation of 1-hydroxy ethyl radical [88,89]. These ROS oxidize cellular DNA, RNA and proteins and initiate membrane lipid peroxidation leading to production of inflammatory mediators and the depletion of the antioxidant defense system causing cellular oxidative stress [90,91]. Controlling the level of ROS levels is linked to cytoprotection and cell survival. Induction of cytoprotective enzymes in response to increased ROS levels is regulated at transcriptional level. This transcriptional response is mediated by *cis*-acting element in the promoter regions of cytoprotective genes, which is referred to as antioxidant response element (ARE). Initially, ARE sequences were defined with two detoxifying enzymes, glutathione S-transferase A2 and NADPH:quinone oxidoreductase [92]. In addition to transcriptional activation of genes, ARE-mediated regulation is also responsible for low-level basal or constitutive expression of several other genes unrelated to oxidative stress. Activation of genes via ARE involves the binding of nuclear factor erythroid-2-related factor-2 (Nrf2). Nrf2 resides in cytoplasm in an inactive form and, upon activation by ROS, translocates to the nucleus to activate genes from ARE [93,94]. Activation of Nrf2 is observed with ethanol exposure [95,96]. Nrf2 knockout mice exposed chronically with alcohol exhibited increased mortality compared with control. The toxic metabolite, acetaldehyde, level was increased due to decreased acetaldehyde metabolism in Nrf2 knockout mice chronically fed with alcohol [97]. In addition, steatosis and inflammation mediated by Kupffer cells was observed after ethanol exposure to Nrf2 knock out mice.

Alcohol exposure causes HIF-1α activation and CYP2E1 potentiates HIF-1α activation in vivo [98,99]. Activation of hypoxia-inducible transcription factor (HIF) is being observed in hypoxic and increased ROS state [100]. A number of genes linked to various cell activities have been found to be upregulated by HIF. HIF binds to the hypoxia response element (HRE) after localization to the nucleus. A genome-wide search for the sequences around HRE indicated no requirement for specific sequences around HRE. It has been observed in studies of HRE elements in the promoter regions of various genes regulated by HIF that HRE is predominantly found in −500 bps regions [101]. Oxidative stress, especially ROS can activate HIF [100]. In addition to direct interaction with HRE sequences, HIF interacts with other transcription factors to regulate the gene transcription. Acute and chronic ethanol feeding to mice results into increased hepatic HIF-1α and HIF-2α [102]. The role of HIF-1α in liver steatosis and inflammation is controversial. Opposing results with HIF-1α hepatocyte-specific knockout mice had been observed with 6% ethanol exposure for 4 weeks. In one study, ethanol caused increased steatosis compared to control while in another study, mice were protected [102,103,104].

Another family of transcription factor, forkhead box, class O (FoxO) plays a critical role in antioxidant and liver cell death responses. Mammals possess four FoxO family members, which includes FoxO1, FoxO3, FoxO4 and FoxO6 [105]. The activity of FoxO is regulated by redox, especially the ROS level, which includes posttranslational modification. It has been demonstrated that FoxO3 possesses pro-apoptotic function in hepatocytes exposed to excessive oxidative stress [106]. In addition, nutrient deprivation-mediated activation of AMPK causes FoxO3 activation specific phosphorylation [107]. In addition, acute ethanol treatment results in FoxO3 retention in the nucleus after Akt-dependent phosphorylation [108]. Due to its regulation by ROS and phosphorylation mediated activation, the toxicity of ethanol was evaluated in FoxO3-deficient mice. The FoxO3-deficient mice developed hepatosteatosis, steatohepatitis and liver necrosis as evidenced by increased alanine aminotransferase level in blood [109]. Another transcription factor, activator protein-1 (AP-1), is activated in rat liver sinusoidal cells and human endothelial cells by short term ethanol exposure. Chronic alcohol exposure in rats also enhanced AP-1 activity [110]. Activated AP-1 increases expression of RANTES and can contribute to ethanol-mediated inflammation [111].

### Chronic Alcohol Intake and Hypertension

Chronic alcohol exposure is linked to hypertension while moderate drinking is linked to beneficial effects such as improvement in cardiovascular risk factor, including high-density lipoprotein cholesterol and inhibitors of thrombosis. Continued consumption of more than 2 servings of ethanol (30–50 g) results in a dose-dependent rise in blood pressure. However, acute exposure causes a modest fall in blood pressure [112]. Heavy alcohol consumption (>2 drinks daily) has been shown to be associated with hypertension [113,114,115].

The mechanism of alcohol-mediated hypertension is obscure. The possible mechanism includes but is not limited to an imbalance of the central nervous system, impairment of the baroreceptor, an increase in sympathetic activity, stimulation of the endothelium to release endothelin, inhibition of endothelium-dependent nitric-oxide production and stimulation of the rennin-angiotensin-aldosterone system, etc. [116]. Lowering of the alcohol-induced hypertension in rats has been obtained by dexamethasone treatment, which abrogates activation of the sympathetic nervous system responsible for releasing corticotrophin-releasing hormone [117]. In another study, an ethanol-induced increase in circulating vasopressin in experimental rats has been implicated in hypertension [118]. Antihypertensive drugs antagonizing renin and angiotensin II binding to its type I receptor are shown to offer protection against alcohol-induced responses in cultured human endothelial cells, suggesting the possible involvement of renin-angiotensin-aldosterone system (RAAS) [119].

The renin-angiotensin-aldosterone system (RAAS) plays a significant role in the regulation of blood pressure. The octapeptide, angiotensin-II (Ang II) is a potent vasopressor molecule released by proteolytic cleavage of the precursor molecule, angiotensinogen (AGT) by renin and angiotensin-I converting enzyme (ACE). The source of circulating AGT is primarily the liver. The inhibition of renin-angiotensin system in experimental animals and clinical studies has proven to be effective in lowering blood pressure in hypertension. The circulating plasma concentration of AGT is close to the Michaelis-Menten (*Kd*) constant of the aspartyl protease, renin [120]. Therefore, alterations in circulating AGT level can cause corresponding changes in blood Ang-II levels. A rise in blood AGT levels will lead to a parallel increase in the formation of active peptides. Increased levels of Ang-II have been shown to cause hypertension. Recent studies in this field have demonstrated a direct link between blood AGT level and blood pressure [121]. Higher blood AGT levels have been demonstrated in hypertensive subjects and their off springs as compare to normotensives [122]. Expression of RAS in multiple body organs, such as kidney, heart, placenta, brain and adrenals are examples of organ-specific blood pressure regulation [123]. Overexpression of human AGT in transgenic animals results in blood pressure elevation, while AGT gene knockout reduces blood pressure in mice [124]. Increasing the copy numbers of AGT in mice results in successive increases in blood pressure [125]. Expression of antisense AGT mRNA causes a profound reduction in blood pressure in hypertensive rats [126]. These studies demonstrate that changes in blood AGT level can quantitatively alter blood pressure. Similar studies duplicating angiotensin-I converting enzyme (ACE) gene in mice led to an increase in blood ACE level, but no quantitative change in blood pressure [127]. Therefore, human studies and experimental models support the role of AGT in human hypertension.

The effect of alcohol exposure on human angiotensinogen levels is not known. However, it has been observed that chronic alcohol exposure followed by binge alcohol exposure causes increased mRNA levels of AGT in mice [128]. Increased levels of mRNA of AGT in mice have been observed in HIGA mice (high immunoglobulin A transgenic mice), which possess increased ROS levels [129]. Formation of increased Ang II can be anticipated from increased synthesis and secretion of angiotensinogen. Recent studies have demonstrated a significant increase in blood and aortic angiotensin II levels after alcohol exposure to rats [130,131]. Prolonged elevation of serum angiotensin converting enzyme (ACE) activity in alcoholics suggests elevated angiotensin II levels due to activation of ACE activity [132]. Sustained RAAS activation with progressive increases in plasma angiotensin II levels, renin activity, left ventricular ACE activity, and left ventricular myocyte Ang II and AT1 receptor expression in dogs have been observed with alcohol exposure [133]. The studies from our laboratory have indicated increased angiotensinogen synthesis and secretion from human hepatocytes after ethanol exposure [134,135]. The mechanism involving RAAS is more likely implicated in alcohol-induced hypertension.

## 5. Summary and Conclusions

Indeed, the metabolic products of ethanol (acetaldehyde and ROS) activate the transcription factors such as NF-κB, HIF and AP-1. Activation of NF-κB in turn leads to activation of the macrophages and Kuppfer cells of the liver. The liver lipogenic program may be activated via SREBPs, which are also activated in ethanol exposure. Established research indicates that the transformation of the liver towards lipid accumulation may occur due to SREBP and possibly other liver-specific transcription factors including PPARα whose activity is decreased resulting into decreased fatty acid utilization.
